# (4*R*)-4-(2-Allyl-2*H*-1,2,3-triazol-4-yl)-1,2-*O*-isopropyl­idene-l-threose

**DOI:** 10.1107/S1600536808036416

**Published:** 2008-11-13

**Authors:** Sarah F. Jenkinson, Daniel Best, Francis X. Wilson, George W. J. Fleet, David J. Watkin

**Affiliations:** a Department of Organic Chemistry, Chemistry Research Laboratory, Department of Chemistry, University of Oxford, Oxford OX1 3TA, England; b Summit plc, 91 Milton Park, Abingdon, Oxfordshire OX14 4RY, England; c Department of Chemical Crystallography, Chemistry Research Laboratory, Department of Chemistry, University of Oxford, Oxford OX1 3TA, England

## Abstract

X-ray crystallography unequivocally confirmed the structure of the title compound, C_12_H_17_N_3_O_4_, as (4*R*)-4-(2-allyl-2*H*-1,2,3-triazol-4-yl)-1,2-*O*-isopropyl­idene-l-threose.  The absolute configuration was determined by the use of d-glucorono-3,6-lactone as the starting material. The crystal structure consists of hydrogen-bonded chains of mol­ecules running parallel to the *a* axis. There are no unusual packing features.

## Related literature

For related background information on the biotechnological inter­conversion of monosaccharides and other sugars, see: Izumori (2002 ▶, 2006 ▶); Granstrom *et al.* (2004 ▶); Yoshihara *et al.* (2008 ▶); Booth *et al.* (2008 ▶); Jenkinson, Booth, Gullapalli *et al.* (2008 ▶); Jenkinson, Booth, Yoshihara *et al.* (2008 ▶); Gullapalli *et al.* (2007 ▶); Jenkinson, Booth, Best *et al.* (2008 ▶). For related literature, see: Görbitz (1999 ▶).
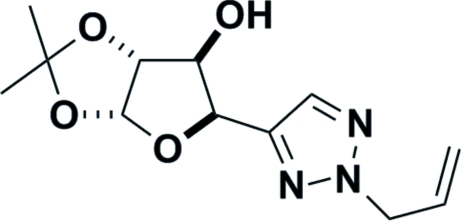


## Experimental

### 

#### Crystal data

C_12_H_17_N_3_O_4_
*M* *_r_* = 267.28
Orthorhombic, 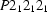

*a* = 5.3959 (2) Å
*b* = 9.6233 (3) Å
*c* = 25.4532 (9) Å
*V* = 1321.69 (8) Å^3^
*Z* = 4
Mo *K*α radiation
μ = 0.10 mm^−1^
*T* = 150 K
0.30 × 0.20 × 0.03 mm

#### Data collection

Nonius KappaCCD diffractometer
Absorption correction: multi-scan (*DENZO*/*SCALEPACK*; Otwinowski & Minor, 1997 ▶) *T*_min_ = 0.82, *T*_max_ = 1.00 (expected range = 0.817–0.997)
9466 measured reflections
1528 independent reflections
1194 reflections with *I* > 2σ(*I*)
*R*_int_ = 0.096

#### Refinement

*R*[*F* ^2^ > 2σ(*F* ^2^)] = 0.041
*wR*(*F* ^2^) = 0.098
*S* = 0.93
1528 reflections
172 parameters
H-atom parameters constrained
Δρ_max_ = 0.32 e Å^−3^
Δρ_min_ = −0.33 e Å^−3^

### 

Data collection: *COLLECT* (Nonius, 2001 ▶); cell refinement: *DENZO*/*SCALEPACK* (Otwinowski & Minor, 1997 ▶); data reduction: *DENZO*/*SCALEPACK*; program(s) used to solve structure: *SIR92* (Altomare *et al.*, 1994 ▶); program(s) used to refine structure: *CRYSTALS* (Betteridge *et al.*, 2003 ▶); molecular graphics: *CAMERON* (Watkin *et al.*, 1996 ▶); software used to prepare material for publication: *CRYSTALS*.

## Supplementary Material

Crystal structure: contains datablocks global, I. DOI: 10.1107/S1600536808036416/lh2725sup1.cif

Structure factors: contains datablocks I. DOI: 10.1107/S1600536808036416/lh2725Isup2.hkl

Additional supplementary materials:  crystallographic information; 3D view; checkCIF report

## Figures and Tables

**Table 1:** Hydrogen-bond geometry (Å, °)

*D*—H⋯*A*	*D*—H	H⋯*A*	*D*⋯*A*	*D*—H⋯*A*
O11—H111⋯O8^i^	0.88	1.95	2.822 (4)	170

Symmetry code: (i) 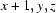.

**Fig. 1.**
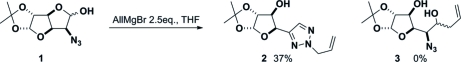
Synthetic Scheme

**Fig. 2.**
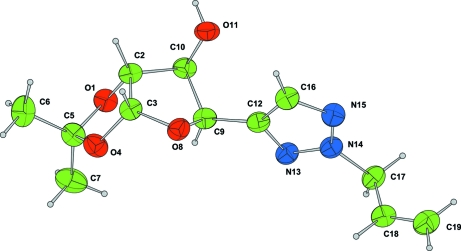
The title compound with displacement ellipsoids drawn at the 50% probability level. H atoms are shown as spheres of arbitary radius.

**Fig. 3.**
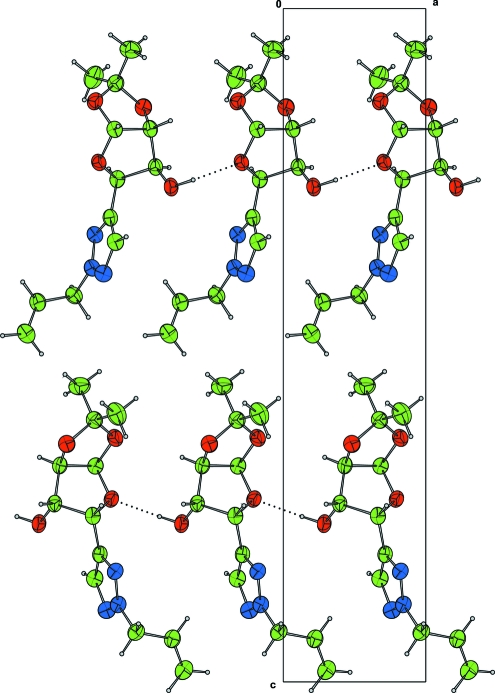
Packing diagram for the title compound projected along the b-axis. Hydrogen bonds are indicated by dotted lines.

## supplementary crystallographic information

### Comment

The process for the biotechnological interconversion of monosaccharides developed by Izumori (Izumori, 2002; Izumori, 2006; Granstrom *et al.*, 2004), has been seen to be generally applicable to other sugar derivatives such as 1-deoxy sugars (Yoshihara *et al.*, 2008; Booth *et al.* 2008; Jenkinson, Booth, Gullapalli *et al.*, 2008; Jenkinson, Booth, Yoshihara *et al.*, 2008; Gullapalli *et al.*, 2007). To evaluate the applicability of this process to 2-deoxy sugars and their derivatives a variety of carbon chain extension reactions were investigated, for example, addition of lithium *tert*-butyl acetate to sugar lactones (Jenkinson, Booth, Best *et al.*, 2008) or addition of allyl magnesium bromide to an aldose.

Reaction of lactol 1 (Fig. 1) with 2.5 equivalents of allyl magnesium bromide generated a single isolable product along with recovered starting material. X-ray crystallography identified the compound as 4*R*-4-(2-allyl-2*H*-1,2,3-triazole-4-yl)-1,2-*O*-isopropylidene-L-threose 2 (Fig. 2) rather than the anticipated addition product 3. The crystal structure was seen to consist of alternating chains of hydrogen-bonded molecules running parallel to the *a*-axis (Fig. 3).Only classic intermolecular hydrogen bonding has been considered. The absolute configuration was determined from the starting material.

### Experimental

The title compound was recrystallized by vapour diffusion from a mixture of diethyl ether and cyclohexane: m.p. 361–364 K; [α]_D_^25^ -13.9 (*c*, 0.69 in CHCl_3_).

### Refinement

In the absence of significant anomalous scattering, Friedel pairs were merged and the absolute configuration was assigned from the starting material.

The relatively large ratio of minimum to maximum corrections applied in the multiscan process (1:1.22) reflect changes in the illuminated volume of the crystal. Changes in illuminated volume were kept to a minimum, and were taken into account (Görbitz, 1999) by the multi-scan inter-frame scaling (*DENZO*/*SCALEPACK*, Otwinowski & Minor, 1997).

The H atoms were all located in a difference map, but those attached to carbon atoms were repositioned geometrically. The H atoms were initially refined with soft restraints on the bond lengths and angles to regularize their geometry (C—H in the range 0.93–0.98, O—H = 0.82 Å) and *U*_iso_(H) (in the range 1.2–1.5 times *U*_eq_ of the parent atom), after which the positions were refined with riding constraints.

### Crystal data

**Table:**

C_12_H_17_N_3_O_4_	*F*_000_ = 568
*M**_r_* = 267.28	*D*_x_ = 1.343 Mg m^−^^3^
Orthorhombic, *P*2_1_2_1_2_1_	Mo *K*α radiation λ = 0.71073 Å
Hall symbol: P 2ac 2ab	Cell parameters from 1500 reflections
*a* = 5.3959 (2) Å	θ = 5–26º
*b* = 9.6233 (3) Å	µ = 0.10 mm^−^^1^
*c* = 25.4532 (9) Å	*T* = 150 K
*V* = 1321.69 (8) Å^3^	Plate, colourless
*Z* = 4	0.30 × 0.20 × 0.03 mm

### Data collection

**Table:**

Nonius KappaCCD diffractometer	1194 reflections with *I* > 2σ(*I*)
Monochromator: graphite	*R*_int_ = 0.096
*T* = 150 K	θ_max_ = 26.0º
ω scans	θ_min_ = 5.3º
Absorption correction: multi-scan(DENZO/SCALEPACK; Otwinowski & Minor, 1997)	*h* = −6→6
*T*_min_ = 0.82, *T*_max_ = 1.00	*k* = −11→11
9466 measured reflections	*l* = −30→31
1528 independent reflections	

### Refinement

**Table:**

Refinement on *F*^2^	Hydrogen site location: inferred from neighbouring sites
Least-squares matrix: full	H-atom parameters constrained
*R*[*F*^2^ > 2σ(*F*^2^)] = 0.041	*w* = 1/[σ^2^(*F*^2^) + (0.04*P*)^2^ + 0.59*P*], where *P* = [max(*F*_o_^2^,0) + 2*F*_c_^2^]/3
*wR*(*F*^2^) = 0.098	(Δ/σ)_max_ = 0.0001
*S* = 0.93	Δρ_max_ = 0.32 e Å^−^^3^
1528 reflections	Δρ_min_ = −0.33 e Å^−^^3^
172 parameters	Extinction correction: None
Primary atom site location: structure-invariant direct methods	

### Fractional atomic coordinates and isotropic or equivalent isotropic displacement parameters (Å^2^)

**Table:**

	*x*	*y*	*z*	*U*_iso_*/*U*_eq_	
O1	1.0189 (4)	0.60546 (19)	0.14662 (7)	0.0395	
C2	1.0670 (5)	0.7244 (3)	0.17865 (11)	0.0366	
C3	0.8174 (6)	0.8008 (3)	0.17938 (10)	0.0374	
O4	0.6798 (4)	0.7427 (2)	0.13781 (7)	0.0436	
C5	0.8248 (6)	0.6390 (3)	0.11107 (11)	0.0407	
C6	0.9294 (7)	0.7022 (4)	0.06094 (12)	0.0579	
C7	0.6697 (7)	0.5117 (4)	0.10209 (15)	0.0608	
O8	0.7063 (4)	0.7727 (2)	0.22853 (7)	0.0394	
C9	0.8362 (6)	0.6572 (3)	0.25347 (11)	0.0371	
C10	1.1041 (5)	0.6779 (3)	0.23535 (11)	0.0371	
O11	1.2131 (4)	0.7866 (2)	0.26506 (8)	0.0425	
C12	0.7862 (6)	0.6641 (3)	0.31060 (11)	0.0359	
N13	0.6792 (5)	0.5578 (2)	0.33594 (9)	0.0372	
N14	0.6586 (5)	0.6032 (2)	0.38514 (9)	0.0372	
N15	0.7386 (5)	0.7336 (2)	0.39351 (9)	0.0406	
C16	0.8223 (6)	0.7724 (3)	0.34651 (11)	0.0396	
C17	0.5321 (6)	0.5246 (3)	0.42598 (12)	0.0404	
C18	0.2777 (6)	0.5809 (3)	0.43621 (12)	0.0439	
C19	0.1964 (7)	0.6160 (3)	0.48272 (12)	0.0499	
H21	1.2090	0.7805	0.1665	0.0468*	
H31	0.8420	0.9045	0.1748	0.0468*	
H61	0.7897	0.7321	0.0390	0.0897*	
H62	1.0308	0.6330	0.0433	0.0901*	
H63	1.0262	0.7815	0.0727	0.0903*	
H73	0.5330	0.5360	0.0790	0.0953*	
H72	0.7699	0.4402	0.0859	0.0956*	
H71	0.6044	0.4809	0.1356	0.0950*	
H91	0.7757	0.5674	0.2382	0.0502*	
H101	1.1990	0.5896	0.2360	0.0485*	
H161	0.8989	0.8595	0.3389	0.0499*	
H171	0.5156	0.4288	0.4138	0.0506*	
H172	0.6324	0.5300	0.4584	0.0502*	
H181	0.1730	0.5914	0.4060	0.0573*	
H192	0.0314	0.6524	0.4857	0.0646*	
H191	0.3038	0.6046	0.5128	0.0648*	
H111	1.3679	0.7927	0.2545	0.0633*	

### Atomic displacement parameters (Å^2^)

**Table:**

	*U*^11^	*U*^22^	*U*^33^	*U*^12^	*U*^13^	*U*^23^
O1	0.0413 (12)	0.0331 (10)	0.0440 (11)	0.0027 (10)	−0.0005 (10)	−0.0048 (9)
C2	0.0328 (14)	0.0335 (15)	0.0434 (16)	−0.0013 (13)	0.0025 (13)	−0.0016 (13)
C3	0.0372 (14)	0.0345 (14)	0.0406 (15)	0.0007 (14)	0.0016 (14)	0.0007 (13)
O4	0.0369 (10)	0.0492 (12)	0.0447 (11)	0.0076 (11)	−0.0044 (10)	−0.0066 (10)
C5	0.0407 (16)	0.0378 (15)	0.0436 (16)	0.0052 (15)	−0.0022 (15)	−0.0015 (13)
C6	0.070 (2)	0.058 (2)	0.0457 (18)	0.0116 (19)	0.0039 (18)	0.0051 (17)
C7	0.057 (2)	0.0484 (19)	0.077 (2)	−0.007 (2)	−0.013 (2)	−0.0080 (18)
O8	0.0321 (10)	0.0450 (11)	0.0411 (10)	0.0061 (10)	0.0042 (9)	0.0053 (9)
C9	0.0349 (15)	0.0313 (14)	0.0451 (17)	0.0007 (13)	−0.0013 (14)	0.0035 (12)
C10	0.0315 (15)	0.0367 (15)	0.0430 (16)	0.0022 (12)	0.0012 (13)	−0.0064 (14)
O11	0.0292 (10)	0.0498 (11)	0.0486 (11)	−0.0048 (10)	0.0023 (9)	−0.0075 (10)
C12	0.0324 (14)	0.0326 (13)	0.0427 (15)	0.0006 (13)	0.0015 (14)	0.0005 (12)
N13	0.0381 (13)	0.0328 (12)	0.0408 (13)	−0.0013 (12)	0.0040 (12)	−0.0010 (10)
N14	0.0380 (13)	0.0320 (12)	0.0415 (13)	−0.0020 (12)	0.0021 (12)	0.0009 (11)
N15	0.0456 (14)	0.0334 (12)	0.0429 (13)	−0.0006 (12)	0.0012 (11)	−0.0013 (11)
C16	0.0408 (15)	0.0332 (14)	0.0447 (16)	0.0013 (15)	0.0017 (14)	0.0009 (13)
C17	0.0420 (16)	0.0359 (15)	0.0433 (17)	0.0005 (14)	0.0062 (14)	0.0027 (14)
C18	0.0396 (17)	0.0435 (16)	0.0486 (17)	−0.0038 (15)	0.0027 (15)	0.0003 (15)
C19	0.0482 (18)	0.0462 (17)	0.0552 (19)	−0.0032 (18)	0.0095 (18)	−0.0042 (15)

### Geometric parameters (Å, °)

**Table:**

O1—C2	1.429 (3)	C9—C12	1.481 (4)
O1—C5	1.421 (4)	C9—H91	1.003
C2—C3	1.535 (4)	C10—O11	1.419 (3)
C2—C10	1.524 (4)	C10—H101	0.992
C2—H21	0.987	O11—H111	0.879
C3—O4	1.409 (3)	C12—N13	1.340 (3)
C3—O8	1.414 (3)	C12—C16	1.400 (4)
C3—H31	1.013	N13—N14	1.331 (3)
O4—C5	1.439 (3)	N14—N15	1.344 (3)
C5—C6	1.522 (4)	N14—C17	1.456 (4)
C5—C7	1.501 (4)	N15—C16	1.332 (4)
C6—H61	0.982	C16—H161	0.954
C6—H62	0.972	C17—C18	1.498 (4)
C6—H63	0.973	C17—H171	0.977
C7—H73	0.972	C17—H172	0.988
C7—H72	0.968	C18—C19	1.307 (4)
C7—H71	0.969	C18—H181	0.959
O8—C9	1.459 (3)	C19—H192	0.960
C9—C10	1.531 (4)	C19—H191	0.967
			
C2—O1—C5	108.4 (2)	C10—C9—C12	117.5 (3)
O1—C2—C3	103.4 (2)	O8—C9—H91	109.4
O1—C2—C10	109.2 (2)	C10—C9—H91	107.6
C3—C2—C10	104.2 (2)	C12—C9—H91	111.1
O1—C2—H21	113.6	C9—C10—C2	101.5 (2)
C3—C2—H21	115.0	C9—C10—O11	109.1 (2)
C10—C2—H21	110.8	C2—C10—O11	110.0 (2)
C2—C3—O4	105.3 (2)	C9—C10—H101	111.8
C2—C3—O8	106.9 (2)	C2—C10—H101	109.6
O4—C3—O8	111.4 (2)	O11—C10—H101	114.1
C2—C3—H31	110.9	C10—O11—H111	106.3
O4—C3—H31	112.0	C9—C12—N13	121.1 (2)
O8—C3—H31	110.2	C9—C12—C16	130.5 (3)
C3—O4—C5	110.1 (2)	N13—C12—C16	108.3 (2)
O4—C5—O1	104.9 (2)	C12—N13—N14	103.8 (2)
O4—C5—C6	108.8 (2)	N13—N14—N15	115.4 (2)
O1—C5—C6	110.6 (3)	N13—N14—C17	122.7 (2)
O4—C5—C7	109.5 (3)	N15—N14—C17	121.5 (2)
O1—C5—C7	108.8 (2)	N14—N15—C16	103.2 (2)
C6—C5—C7	113.9 (3)	C12—C16—N15	109.3 (3)
C5—C6—H61	108.0	C12—C16—H161	125.6
C5—C6—H62	108.8	N15—C16—H161	125.1
H61—C6—H62	111.7	N14—C17—C18	111.5 (2)
C5—C6—H63	104.7	N14—C17—H171	107.9
H61—C6—H63	110.9	C18—C17—H171	108.2
H62—C6—H63	112.2	N14—C17—H172	108.2
C5—C7—H73	108.6	C18—C17—H172	109.7
C5—C7—H72	109.5	H171—C17—H172	111.4
H73—C7—H72	109.7	C17—C18—C19	123.9 (3)
C5—C7—H71	108.6	C17—C18—H181	116.0
H73—C7—H71	109.2	C19—C18—H181	120.0
H72—C7—H71	111.1	C18—C19—H192	118.6
C3—O8—C9	109.1 (2)	C18—C19—H191	119.2
O8—C9—C10	102.9 (2)	H192—C19—H191	122.2
O8—C9—C12	107.8 (2)		

### Hydrogen-bond geometry (Å, °)

**Table:**

*D*—H···*A*	*D*—H	H···*A*	*D*···*A*	*D*—H···*A*
C16—H161···O1^i^	0.95	2.44	3.322 (4)	154
C17—H171···O4^ii^	0.98	2.46	3.362 (4)	154
O11—H111···O8^iii^	0.88	1.95	2.822 (4)	170

Symmetry codes: (i) −*x*+2, *y*+1/2, −*z*+1/2; (ii) −*x*+1, *y*−1/2, −*z*+1/2; (iii) *x*+1, *y*, *z*.
